# Consumer Advertising Can Be Misleading

**DOI:** 10.1371/journal.pmed.0030119

**Published:** 2006-02-28

**Authors:** Karl Rickels

**Affiliations:** **1**University of PennsylvaniaPhiladelphia, PennsylvaniaUnited States of America

The Essay by Lacasse and Leo [ 1] demonstrates clearly how consumer advertising—in all fields of medicine, not only psychiatry—can at the least be misleading, making patients choose treatments that may not be the best choice in each particular circumstance. I agree with the authors that the “serotonin” hypothesis does not fully explain the mechanism of the serotonin reuptake inhibitors (SSRIs) in the treatment of anxiety disorders and depression.

In my clinical treatment of patients, consumer advertising is not only not helpful but often causes significant management problems. In addition, consumer advertising only focuses on expensive, patented medications and not on equally good generic alternatives. A good example is the consumer advertising of “the purple pill,” Nexium, while generic Prilosec, equally effective in almost all patients, is not advertised.

Let's prohibit all consumer advertising of patented medications. It will save physicians much headache, and patients or their insurers a great deal of money.
